# Military Amputations. I. A Reversion to Pre-Listerian Methods

**Published:** 1916-05-27

**Authors:** 


### MILITARY AMPUTATIONS.

### I. A Reversion to Pre-Listerian Methods.

When the war began it is fairly safe to say that practically all the operators in the Royal Army Medical Corps set out to practise amputations, in such cases as they were deemed necessary, on the usual lines of surgical teaching as expounded in the leading text-books of operative surgery. Before many months had elapsed, a general unanimity had been reached, as a result of bitter experience, among those surgeons who were in a position to oversee their own and other peoples' results, to the effect that the technique of amputations in civil practice is very largely inapplicable to the exigencies of military practice in this campaign. The factor which has caused this change of attitude is the same as that which has led to so many other deviations from civilian medical routine—namely, the very heavily infected nature of the wounds and the frequency of gangrene and other troubles.

### Amputations in Civil Practice.

In ordinary peace-time practice amputations are becoming quite rare operations: in so many of the conditions for which they were in pre-Listerian days resorted to, surgeons are now able to save the patient's limb as well as his life. When for good and sufficient reason in civilian practice an amputation becomes unavoidable, one or other of various ingenious "flap" operations is nearly always employed in place of the "circular" amputations favoured by our great-grandfathers. Moreover, the separation of the limb in nine cases out of ten is effected at one or other of the "sites of election," which are certain situations where a flap operation is readily practicable, and the resulting stump is especially suitable for the fitting of an artificial limb afterwards. When the operation is completed, whether it be a "flap" or a "circular," or a combination of the two, such as the "racquet" operation, the wound is carefully sutured so as to ensure primary union, usually with one or more drainage apertures to relieve tension for the first day or two.

### Reversion to Pre-Listerian Methods.

How different is the military amputation of the present moment! Flap operations are very rarely done indeed, for the reason that the flaps interfere with the free drainage which is essential in these heavily infected wounds; moreover, the flaps themselves are found to slough, or even to become themselves gangrenous. No suturing takes place in the majority of cases, for exactly the same reasons. Circular amputations are in favour, and it is necessary to leave the whole wound open—a spectacle revolting alike to æsthetic susceptibilities and to all the surgical ideals that have sprung up since the Listerian epoch began. In order to counteract the deadly microbes of "gas gangrene" many surgeons have treated their patients out of doors (in suitable weather), with no dressings on the raw stump of the limb: this plan has in view the maximum oxygenation of the surface of the wound, with correspondingly unfavourable conditions for the anaërobic organisms of gangrene. Primary union is, of course, unknown when these methods are used.

The adoption of these pre-Listerian types of amputation was forced upon the surgeons by the stern logic of events. At the beginning of the campaign flap amputations with sutures were universal, or almost so. The present writer recalls three or four which he did with a considerable amount of self-satisfaction. Subsequent experience has led him to wonder what the fate of his unfortunate patients was later on; for owing to the fact that they were treated in a field ambulance, in one case actually under fire, the patients passed out of his hands within twenty-four hours of being operated upon. Probably they got gangrene, and either perished miserably or suffered re-amputation at a higher level.

### The Passing of the Flap Operation.

Wisdom, however, was acquired by bitter experience, and by the end of 1914 flap operations were already discredited in France, though it was not always easy to get this view accepted by newly arrived surgeons with no experience of the novel conditions. Even as late as the autumn of 1915 a certain surgeon of fairly considerable parts and experience, who came over to France, flatly declined to believe in the necessity for circular amputations and open wounds, and insisted on following out the customary routine of home practice: he was soon converted. The writer recalls a case which impressed him strongly at the time, and since it is typical of what happens when flap operations are adopted, it may be worth recounting. He entered an operating-room just as a young and capable surgeon fresh from England was about to tackle a badly smashed ankle-joint by amputating two or three inches above it. He strongly advised the operator to remove much more than he was planning to: to operate, in fact, at the site of election, three inches below the knee. This was about the time when the flap operations were in process of being discarded, but it was thought that by operating through tissues so far removed from infection the flap amputation (Farabœuf's in this case) was justifiable: the wound was sutured. Four days later both flaps were black and the patient was obviously very septic: re-amputation above the knee by a circular operation without sutures saved the man's life. This was the last flap operation which was performed (in France) by the writer or anyone who asked his opinion and advice about an amputation case. A further point is that owing to the severe comminution of so many gun-shot fractures, amputations at sites of election are in very many cases impossible. For this reason, as well as through the disuse of flap operations, the after-treatment of these cases, especially in respect of the provision of artificial limbs, presents many difficult problems. How these are being met, and how they can be anticipated to some extent by the surgeon, will be discussed in a future article.

